# A new digital tool for radiographic bone level measurements in longitudinal studies

**DOI:** 10.1186/s12903-015-0092-9

**Published:** 2015-09-08

**Authors:** Hans R Preus, Gerald Ruiner Torgersen, Odd Carsten Koldsland, Bjørn Frode Hansen, Anne Merete Aass, Tore Arne Larheim, Leiv Sandvik

**Affiliations:** Department of Periodontology, Institute of Clinical Odontology, Faculty of Dentistry, University of Oslo, PO 1109 Blindern, 0317 Oslo, Norway; Department of Maxillofacial Radiology, Institute of Clinical Odontology, Faculty of Dentistry, University of Oslo, PO 1109 Blindern, 0317 Oslo, Norway

## Abstract

**Background:**

The reproducibility of measurements on radiographs is influenced by the techniques by which the images as well as the measurements are obtained. Thus, bias resulting from errors in the image and/or image examinations at two points in time may result in wrongful registrations of true biological or pathological changes. The aim of the present study was to propose and evaluate an indirect radiological examination technique, by which bias, when measuring radiographic bone level, could be substantially reduced as compared to the technique using direct mm measurements.

**Methods:**

A plugin to ImageJ was designed to reduce bias when measuring bone loss on radiographic images. In human dry mandibles, radiographic images of 20 teeth were obtained parallel with the tooth axis (alpha = 0) and at an angle of 30° deviation. The direct technique of measuring radiographic bone level (RBL) and the indirect, length-adjusted RBL were registered by four researchers in a double blinded fashion.

**Results:**

When mean RBL measured at 0° angle was 7.0 mm, the corresponding mean RBL measured at 30° angle was 7.8 mm, signifying an 11.4 % increase (*p* = 0.032), whereas the mean length-adjusted RBL increased by 0.6 % (*p* = 0.9).

**Conclusions:**

This study showed that the use of the original, direct technique (ImageJ) resulted in markedly biased radiographic bone level at 30° angle, while the proposed indirect length-adjusted technique (ImageJ plugin) did not.

## Background

Periodontal destruction is most frequently described in mm periodontal pocket depth (PD) and clinical attachment level (CAL) [1–3]. However, despite being the most common way to convey periodontal disease, these soft-tissue measures are considered inaccurate, surrogate parameters reflecting an unpredictable combination of gingivitis and periodontitis as well as being variably affected by the examiners skill, reliability and technique [3–5].

Radiographic examination is an alternative to PD and CAL in evaluating periodontal bone level, and is considered a more reliable estimate of disease experience. A decrease or increase in the alveolar bone level at a given site over a period of time may be regarded as progression or regression of the disease process at that site [6]. However, registrations made on radiographs are influenced by the techniques by which the images, as well as the measurements, are obtained [7, 8]. A long cone “paralleling technique” [9], with a standardized receptor holder [10], is recommended for obtaining the radiographs, and it has been suggested that the direct method (in mm) should be used when investigating the susceptibility of different teeth to periodontal breakdown in clinical trials of comparative nature [10, 11].

However, researchers have warned that image distortions of the object (tooth) appear if there is deviation from the “paralleling technique” [12, 13]. This deviation is especially evident, as it is inevitable, in the upper jaw where the palatal anatomy prevents the ideal positioning of the receptor [14, 15]. In a comprehensive study, Roeder and co-workers [16] reported mean angulation for central incisors and first molars to be 37° (range 19–56°) and 42.5° (range 26–56°), respectively. Furthermore they showed “foreshortening” of the image of these teeth ranging from 5.4 to 44.1 %.

In longitudinal, clinical studies high reproducibility of registrations is of the essence, and distortion of imaged teeth and surrounding bone on radiographs will result in unreliable measurements of the bone level and tooth length [17, 18] and consequently, give a wrongful impression of loss or gain of bone over time. Researchers are therefore calling for new techniques that could correct such distortions [16].

The purpose of the present experimental study was therefore to propose and test a rapid and indirect, semi-automated radiographic method for periodontal bone level assessments that reduces the distortion of radiographic bone level (RBL) changes caused by varying angles when obtaining the radiographic image.

## Methods

**Fig. 1:**
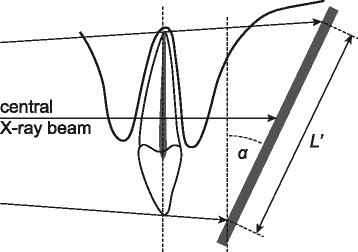
Schematic setup of the angles of projection

**Fig. 2:**
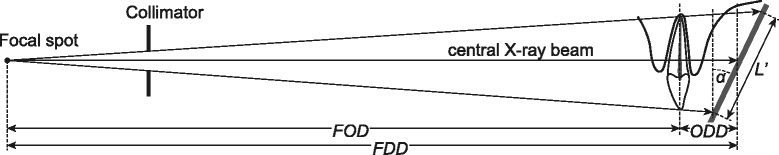
Schematic setup of imaging geometry

**Fig. 3:**
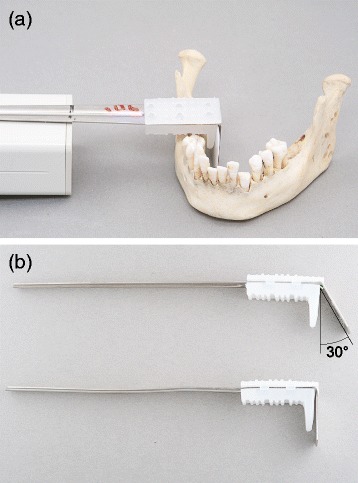
**a.** Experimental setup showing elevated teeth in their sockets and Eggens holder with sensor at α = 0°. **b.** The Eggens holders with regular α = 0° and modified to α = 30°

Seven human dry mandibles bearing 20 (already) loose teeth were used for this experiment. The use of these mandibles, for this experiment and under these conditions, did not require approval from the regional committee for research ethics due to national legislation. The physical lengths of each of the teeth measured by an electronic caliper (Cocraft®, United Kingdom) are shown in Table 1. Mandibles were used in order to control the angle (**α**) between the receptor and the perpendicular-to-the-central X-ray beam (Figs. 1, 2). None of the loose teeth and surrounding bone in these mandibles showed signs of severe periodontal disease as the mean distance from alveolar crest (AC) to the cemento-enamel junction (CEJ) was 1.7 mm. “Severe” or “extensive” periodontitis is reflected by clinical attachment loss (CAL) of more than 5–7 mm [19, 20], which corresponds to a RBL of >7 mm. Since the dry mandibles did not display such degree of bone loss, an artificially reduced bone level was created by placing hard wax in the 20 sockets and replacing the teeth into the wax, allowing a stable marginal bone level of approximately 7 mm (Fig. 3a). One Eggen holder [10] was modified to give the X ray beam – receptor an angle (**α**) of 30°, mimicking a clinical situation [16] (Fig. 3b). A standard Eggen holder, with the receptor in a fixed parallel position with the tooth axis, was used as control (**α** = 0°) (Fig. 3b). The experimental setup is shown in Fig. 3. Consecutive radiographs were obtained in each position (**α** =0° and **α** =30°), of the 20 teeth, by the same operator, scanned and stored. Individually fitted stents were used for securing identical placement of the holders during all exposures of the same area, also securing that the teeth did not sink further into the wax in the bottom of their sockets during the experiment. Radiographic images were obtained by the Soredex Digora Optime Digital imaging plate system film (Soredex, Finland) and a Planmeca intra (Planmeca Oy, Finland) X-ray unit. The images where exported by the Digora for Windows software to 8 bit grey scale Tagged Image File Format (TIFF) files. The calibration information in the image files (i.e. Exif information) were used to obtain the coordinates of the sites. The files where then numbered, randomly reorganized and blinded to the readers.

**Table 1:** The physical length in mm of the 20 teeth with artificially elevated bone level as compared to the length obtained by reading the lengths on the radiographs

Scull No.	Root designation	Root length in mm^a^.	Root length in mm measured on X-rays^b^
1	36M^c^	21.34	21.5.
1	36D^c^	20.35	21.4.
1	35	20.71	21.6.
1	34	20.28	21,7.
1	32	23.36	23,4.
1	43	24.13	25.4.
1	44	21.20	21.3.
1	45	21.19	22.7.
5	37M	20.14	20.4.
5	37D	19.13	19.5.
5	35	21.20	20.8.
5	47M	20.23	22.6.
5	47D	19.52	20.7.
6	43	18.26	18.1.
6	44	18.90	19.5.
7	37M	18.39	19.9.
7	37D	18.17	19.0.
7	35	20.10	20.4.
7	32	23.19	23.2.
7	42	23.75	24.3.
7	45	22.14	22.4.
8	47M	19.06	19.8.
8	47D	18.34	18.9.
9	36M	21.19	21.4.
9	36D	20.26	21.0.
10	33M	18.98	19.7.

^a^Tooth Length measured by Digital caliper (Cocraft, United Kingdom)

^b^Average Tooth length measured on radiographs by examiner 1 (HRP)

^c^ *M* mesial root; *D* distal root

**Fig. 4:**
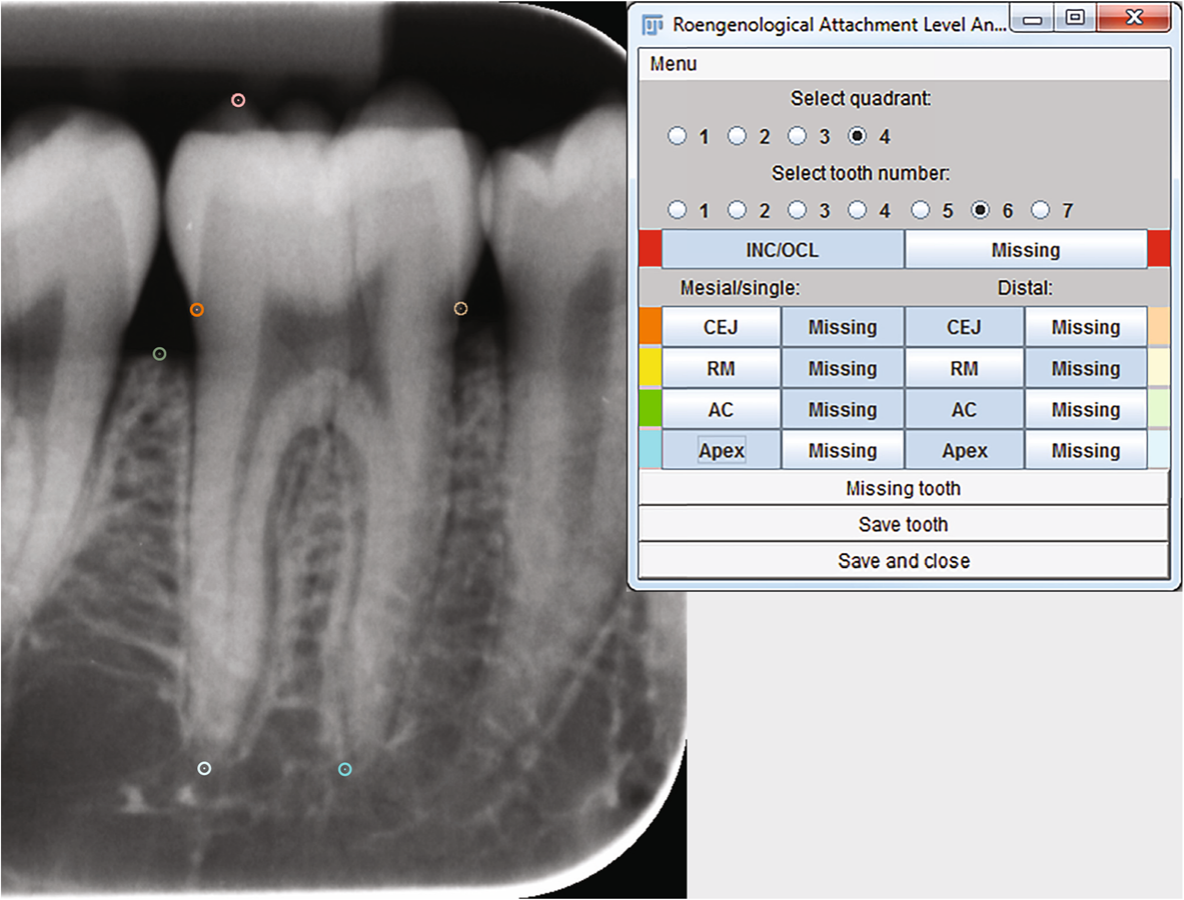
Radiograph with markings and application (plugin) running

**Fig. 5:**
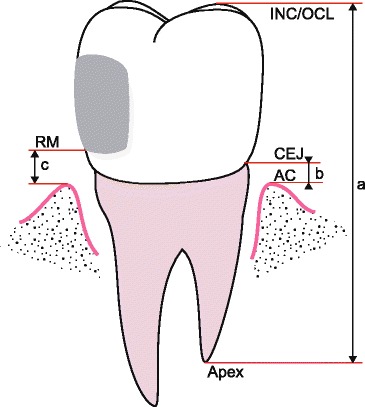
Schematic drawing of the measuring points of the imaged object (tooth). Points are marked on both mesial (M) and distal (D) aspect of image

The latest version of the ImageJ image processing and analysis software program [21] was used for the experiment (ImageJ http://rsbweb.nih.gov/ij/). An application (plugin) to this program was designed (Fig. 4) with the intent to semi-automatically register the ratio between the bone level and the corresponding length of tooth (b/a, Fig. 5) (ImageJ plugin; http://www.odont.uio.no/english/research/projects/periodontal-diseases/). The original ImagJ processing and analysis software program only allows for measuring absolute distances. The measured lengths of the teeth on radiographs are shown in Table 1.

On the digital radiographs points were registered (mouse-clicked) on the incisor edge/summit of crown (INC/OCL), the apex(es) of the root(s) (APEX), as well as the mesial and distal alveolar crests (AC) and cemento-enamel junctions (CEJ) (Fig. 4). For bicuspids and molars, the most elevated cusp on the image was registered, and the distance to the apex(es) was(were) registered from this one, most elevated cusp. The computer application automatically stored the marked coordinates (relative to the upper left corner of the image) and projected them onto an imaginary vertical axis parallel to the vertical dimension of the receptor. In the program the possibility to register restoration margins (RM, Fig. 5) was also designed, although being redundant in the present experiment. The coordinates, unit of length, time and date of measurement were automatically stored in a text file, which could be imported into any spreadsheet or statistical package to calculate length and all possible combinations of distances (Fig. 5) and ratios or percentages - thereof on the imaginary axis. In this study the ratios or percentages of the distance from the CEJ to the AC vs. the length of the tooth (*rel*RBL = b/a, Fig. 5) was the topic of interest.

The radiographs were read blindly twice, by four specialists in periodontology. The examiners were given a duplicate of two sets of digital radiographic images, obtained at **α** 0° and 30°. In one set of images the mesial and distal RBL (b, Fig. 5) were obtained by direct measurements using the traditional ImageJ software program. In the other, the experimental ImageJ plugin was applied, and length adjusted RBL (*rel*RBL) (b/a, Fig. 5) was obtained. In total, 40 sites on 20 radiographs obtained at each **α** (0 and 30°) were read twice with both the direct (ImageJ) and indirect (ImageJ plugin) technique.

### Statistics

When comparing mean score of data on a distance parameter, e.g., bone level, based on radiographs taken at **α**s 0^o^ or 30°, a paired samples *t*-test was used, with 5 % significance level. The statistical analysis was performed by using the statistical software program IBM-SPSS version 18. The Intraclass Correlation Coefficient (ICC) [22] was used to estimate inter- and intra-examiner reliability for both the original (ImageJ) and new method (ImageJ plugin).

## Results

The results from registrations of the radiographic images of tooth length, RBL, and length adjusted RBL (*rel*RBL) (Fig. 5) are shown in Table 2. When mean RBL measured at **α** = 0° was 7.0 mm, the corresponding mean RBL measured at **α** = 30° was 7.8 mm. This 11,4 % increase was statistically significant (*p* = 0.032). Table 2 also shows that the length of the tooth was significantly increased from one image to the next as the angle (**α**) increased from 0° – 30° (*p* < 0.001).

**Table 2:** Length of tooth = INC/OCL - APEX, RBL (Roentgenological Bone Level = AC-CEJ) and *rel* RBL (%RBL/L) in X-rays obtained at 0 and 30 ° angle with artificially increased AL (actual bone level ~ 7 mm)

**α**		0	30 °	*p*-value
INC/OCL – Apex (mm)	mean	22.1	24.7	<0.001
	SD	2.3	2.2	
RBL (mm)	mean	7.0	7.8	0.032
	SD	1.1	1.6	
*rel*RBL (%)	Mean	31.4	31.6	0.903
	SD	3.8	5.3	
N		*N* = 40	*N* = 40	

We defined length-adjusted RBL (*rel*RBL) as RBL divided by the length of the corresponding tooth (L), which can be displayed as a ratio, or as a percentage of the tooth length. The mean length-adjusted RBL (*rel*RBL) increased by only 0.6 % (from 31.4 to 31.6; *p* = 0.9) when measured respectively at 0° and 30° angle.

The ICC for inter- *and* intra-examiner reliability of length of teeth (L) measured by the direct and the indirect method were respectively 0.96 (95 % Cl: 094–0.98) and 0.98 (95 % Cl: 0.96–0.99). The corresponding ICC for RBL were 0.86 (95 % Cl: 0.81–0.89) and 0.94 (95 % Cl: 0.90–0.97) and length–adjusted RBL (*rel*RBL) were 0.82 (95 % Cl: 0.75–0.88) and 0.94 (95 % Cl: 0.90–0.97), respectively.

## Discussion

The obvious distortion problem-especially with radiographs from the maxillary teeth [14]- its confirmation in clinical trials of comparative nature [15, 16], and the call for techniques that could correct for such distortions [15, 16], especially regarding an ongoing longitudinal clinical intervention study [23], prompted the development of the presented digital tool. It is clearly no more than a digital, more versatile version of the Schei ruler [24, 25] and Björn’s technique [26, 27] that at present may be more useful in research, i.e., longitudinal studies where radiographic images are obtained more than once over several years. However, with the developing digital radiographic techniques, hard – and software, it might find its future usefulness to the clinicians as well.

The artificial bone levels, created by elevating the teeth in their sockets relative to the surrounding alveolar bone crest, did not differ from comparative *in vivo* bone levels of severe periodontal disease [19, 20]. Authentic dry skulls are hard to come by and even harder to use for studies due to considerations on ethics and preservation. Therefore, we could not find enough authentic dry mandibles with the desired amount of bone loss, and since removing bone to create an artificial bone loss of 7 mm was considered unethical, an artificial bone level was produced instead by elevating 20 teeth in their sockets. The choice of the angle (**α**) = 30° was made to mimic the clinical situation [16] when obtaining radiographs; an angle that differ 30° between the first and second exposure in a clinical situation is not uncommon. The bone level of 7 mm was chosen since severe [19] and extensive [20] periodontal diseases are described by CAL of >5–7 mm corresponding to a marginal (AC-CEJ) and radiographic bone level (RBL) ≥7 mm.

Correlation to the true length of the teeth corresponded with the general notion that there is a 5–6 % magnification of a tooth projected onto a receptor during regular x-ray imaging (Table 1). The measurements were not corrected for this magnification since all images were obtained with the same personnel, technique and equipment. For obvious reasons, a new technique could not have been evaluated by measurements on regular *in vivo* radiographs since the actual bone level, length - and angles of the teeth in the jaw as well as the angle (α) would have been unknown values, not controlled by the researchers. Correlations to clinical measures were redundant in as much as the present study had to be *ex-vivo* since the aim was to evaluate the indirect radiological examination technique. However, a table has been added to show these values (Table 1).

A pre-study on 12 dry skull mandibles with 35 loose teeth was also performed before the bone level was artificially reduced in 20 of these teeth (described above) and produced practically the same results as those by Roeder et al. [15] (data not shown). However, in the present study, when the true RBL value was 7 mm, as artificially achieved in these dry mandibles, this bias was significant and as large as 0.8 mm (>11,4 %), whereas no significant bias was observed for the length-adjusted RBL (*rel*RBL).

Considering that RBL and *rel*RBL had similar intra - and inter-examiner reliability, these results added to the notion that the indirect registration technique (*rel*RBL) appeared to be a more reliable measure of bone loss than the direct registration technique (RBL). The use of the ICC may be controversial in clinical studies, and especially when the ICC coefficient is in the mid-ranges, since it is not easy to determine if a mid-range ICC score is good or bad. However, in this non-clinical study we deemed ICC as sufficient to examine inter – and intra examiners’ reliability, especially since the ICC was mostly in the high - very high ranges.

There are clinical ways to reduce random or systematic errors during traditional radiographic examinations [28–31], and there are techniques to correct image distortion [13, 14, 32, 33], but these seem less suitable for large-scale, longitudinal studies that goes on for years. Another technique is anterior and molar/premolar bite-wing radiographs. This technique may not be reproducible in patients with severe periodontal disease due to extensive bone destruction preventing the upper and lower jaw bone level to show on the same bite-wing image. Moreover, large receptors frequently cause patient convulsion and evasion reflexes that makes such images difficult to obtain. Thus, standardized peri-apical radiographs seem so far to be the method of choice to monitor bone level in longitudinal clinical interventions trials. However, this sets the stage for the aforementioned distortion errors.

The presented plugin technique finds general application as it may be used for other than periodontal measurements, like oral surgical, cariological and endodontic evaluations and purposes. However, the smaller the object or length measured, the less clinically significant is the distortion problem. Applying the attached formula (Fig. 1) it can be shown that if all objects or lengths measured in a study are below 2 mm, the presented technique is of little value as compared to the direct technique. On the other hand, if all objects are larger than 2 mm, or there is a variation in object size or length from below 2 mm and increasing, the presented technique has been found to be a useful and reliable tool. Moreover, it is still faster to use and less exhausting to the readers. Actually, with the above mentioned limitations, any radiographic measurement, over time, that diagnose changes in bone/tooth/tissue anatomy where the angle **α** (Figs. 1, 2) may vary from examination to examination, may benefit from this technique. However, an adapted protocol/version (point definition) of the plugin should be produced for each purpose. This may be done by visiting the above mentioned website and downloading the plugin (ImageJ plugin; http://www.odont.uio.no/english/research/projects/periodontal-diseases/).

For obvious reasons, it was not possible to test convenience and rapidity of the new technique in a proper scientific manner. However, it should be mentioned that the presented technique seemed faster, and caused less exhaustion among the readers than the original technique. (These are personal observations, although all four readers subscribed to this statement).

## Conclusion

When examining radiographic images for longitudinal changes of the periodontal bone level, the direct technique were markedly biased when the angle between the X-ray beam and the sensor was 30°. This was not observed with the indirect, length-adjusted technique proposed in the present study. Thus the presented, indirect technique seems to be an appropriate radiographic method for longitudinal measurements of the periodontal bone level.

## Abbreviations

AC: Alveolar crest
OCL: Summit of crown
CAL: Clinical attachment level
ODD: Object detector distance
CEJ: Cemento enamel junction
PD: Pocket depth
FOD: Focus object distance
*rel*RBL: Length adjusted Radiographic Bone Level
FDD: Focus detector distance
ICC: Intraclass Correlation Coefficient
RBL: Radiographic bone level
INC: Incisor edge
RM: Restoration margin
L: Length of tooth
TIFF: Tagged image file format
